# Relationships among Physical Fitness, External Loads, and Heart Rate Recovery: A Study on Futsal Players during an Overseas Congested-Weeks Training Camp

**DOI:** 10.5114/jhk/176299

**Published:** 2024-02-17

**Authors:** Yi-Wen Chiu, Rui Miguel Silva, Halil Ibrahim Ceylan, Filipe Manuel Clemente, Francisco Tomás González-Fernández, Yung-Sheng Chen

**Affiliations:** 1 Department of Physical Education, Fu Jen Catholic University, New Taipei City, Taiwan.; 2 Escola Superior Desporto e Lazer (School of Sport and Leisure), Instituto Politécnico de Viana do Castelo (Polytechnic Institute of Viana do Castelo), Viana do Castelo, Portugal.; 3 Sport Physical Activity and Health Research & Innovation Center (SPRINT), Viana do Castelo, Portugal.; 4 Physical Education and Sports Teaching Department, Kazim Karabekir Faculty of Education, Atatürk University, Erzurum, Turkey.; 5 Department of Biomechanics and Sport Engineering, Gdansk University of Physical Education and Sport, Gdańsk, Poland.; 6 Department of Physical Education and Sport, Faculty of Sport Sciences, University of Granada, Granada, Spain.; 7 Department of Exercise and Health Sciences, University of Taipei, Taipei, Taiwan.; 8 Exercise and Health Promotion Association, New Taipei City, Taiwan.; 9 Tanyu Research Laboratory, Taipei, Taiwan.

**Keywords:** match substitution, heart rate responses, match activities, match performance, indoor soccer

## Abstract

This study examined relationships among players’ physical characteristics, match external loads, and heart rate recovery (HRR) during match substitutions in a congested fixture of an overseas futsal training camp. Eleven under-20 national futsal players’ anthropometric characteristics (age, body height, body mass, % fat, and % muscle) and physical fitness [HRmax, VO_2_max, maximal aerobic speed (MAS) during the 30–15 intermittent fitness test (IFT)] were determined. Additionally, locomotion profiles during field play and HRR sitting on the bench were recorded during five matches. A repeated-measures analysis of variance and Pearson's correlation coefficient were used for statistical analysis. The results revealed that the overall observed correlations among anthropometry, body composition, physical fitness, and HRR were inconsistent across all the matches and substitutions. However, the numbers of moderate (1.00–1.99 m/s^2^), moderate-to-high (2.00–2.99 m/s^2^), and high (3.00–50.00 m/s^2^) intensities of acceleration presented negative correlations in the last match (r < −0.76; p < 0.05). HRR during match substitutions may have been influenced by uncontrolled factors across all the match play and recovery. HRR measures may be affected mainly by fatigue caused by the accumulation of accelerations throughout a congested fixture during a congested-schedule of a futsal training camp.

## Introduction

Futsal, recognized as indoor soccer, is characterized by short explosive multiple sprints (usually 1 to 4 s), high-intensity interval activities with numerous accelerations and decelerations, rapid changes of direction, as well as technical and tactical aspects (Spyrou et al., 2020). Elite futsal players spend over 83% of their actual playing time at intensities exceeding 85% of their maximum heart rate (HR_max_) [approximately 170–190 beats per minute (bpm)], and as a result of this high anaerobic work rate (Charlot et al., 2016), tend to burn energy at rates ranging between 16.3 to 18.0 kcal per minute (Wolański et al., 2017). In international matches, futsal players cover a distance of 3–5 km per game with more than 50% of this distance consisting of repeated high-intensity actions occurring every 79 s (exceeding 93% of HR_max_) (Ribeiro et al., 2022), causing blood lactate concentrations to reach up to 21.8 mmol•L^−1^ (Bekris et al., 2022). The above-mentioned studies demonstrate the importance of physical capacity, which is a fundamental prerequisite in futsal (Reinhardt et al., 2020). Also, research findings have indicated that professional futsal players can develop maximum oxygen uptake (VO_2max_) values above 60 ml·kg^−1^•min^−1^, which is crucial for providing faster recovery between efforts or even after exhaustion (Bekris et al., 2022) and handling the physical and physiological demands of the futsal game (Amani-Shalamzari et al., 2020).

The heart rate recovery (HRR) measure is calculated as the rate of a decrease in the HR in the minutes immediately following the cessation of physical exercise (Daanen et al., 2012). Specifically, it involves measuring the HR at specific time intervals during recovery after exercise or physical activity (Daanen et al., 2012). The most common approach is to monitor the HR immediately during recovery, although variations exist where HR recovery is tracked over different time intervals (e.g., 1, 2, or 3 min) (Buchheit, 2014). The rate of the HR decrease is then determined by subtracting the HR at each time interval from the HR at the end of the exercise. The measure expresses this difference as bpm or as a percentage of the peak HR achieved during the exercise (Fecchio et al., 2019). To measure HRR, the participant typically wears an HR monitor during the exercise or activity, and the monitor continues to record HR values during the initial recovery phase.

HRR is recognized as one of the most potent objective indicators that assist in adapting players' training loads and identifying early signs of maladaptation (Campos et al., 2012). HRR is commonly used in team sports to monitor training loads during practices or competitions and to optimize the recovery process most suitably. It can be expressed as the rate at which the HR decreases within minutes following the cessation of physical exercise. This measure encompasses synchronized interplay between the parasympathetic system's reactivation and the sympathetic system's withdrawal (Daanen et al., 2012). An increase in HRR after exercise or a game is widely recognized as a sign of improved aerobic fitness and cardiovascular health in team sports, especially futsal (Daanen et al., 2012). A systematic review highlights a close relationship between HRR and training status, indicating that elite athletes recover faster (Daanen et al., 2012). Similarly, it has been demonstrated that the rate of the HR decline is linked to an advanced aerobic capacity that facilitates and accelerates recovery after anaerobic exertion (Stupnicki et al., 2010).

In futsal, playing consecutive matches (2 to 3 games per week) with very short rest periods during congested game schedules can add a significant load to players' physiological and psychological systems, including the muscular and nervous systems (Ribeiro et al., 2022, 2023). This may lead to the increased likelihood of residual fatigue, risk of injury, significant strength deficits, and reduced physical performance due to decreased time for proper physical recovery (Page et al., 2023). It was found that futsal athletes experienced a decrease in the intensity of their efforts, as measured by %HR_max_, towards the end of a tournament where they played four games over two days (Wolański et al., 2017). The studies mentioned above suggest that fatigue resulting from previous matches significantly impacts players' performance, especially during congested schedules. Thus, sufficient recovery time after an earlier game is widely acknowledged as an essential determinant of futsal success in tournaments (Rahimi et al., 2020). Coaches must use various training strategies and match tactics, including player substitutions, to maintain their athletes' high performance, optimize their workload, and ensure effective recovery during intense weeks (Haller, et al., 2022; Ribeiro et al., 2022).

More player substitutions during congested game schedules will decrease the total game time exposure and can be used as a strategy to reduce players' external and internal loads (i.e., total distance covered and the rating of perceived exertion (RPE) level) (Milanez et al., 2020). According to Drew and Finch (2016), a negative correlation was observed between the RPE and recovery status the day after the competition, suggesting that athletes who experienced a high perceived exertion level tended to have lower recovery indices. Similarly, another study reported that players with higher training loads, VO_2max_, and repeated sprint ability showed higher playing intensity and slower recovery indices during an international futsal tournament played in a congested schedule (Charlot et al., 2016). In light of this information, previous studies have demonstrated that frequent player substitutions during a congested week can allow players to maintain high-intensity activities, prepare physically for offensive and defensive actions, recover more quickly (Clay and Clay, 2014), delay muscle damage and fatigue effects (Ribeiro et al., 2022, 2023), and maintain their performance at its peak throughout the matches during the congested schedule (Bekris et al., 2022).

This study aims to address a notable research gap that stems from the unique challenges faced by futsal players during intense weeks of consecutive matches. While prior studies have underscored the importance of physical fitness, player substitutions, and recovery strategies, little is known about how these factors interplay with the HRR processes. Specifically, this study seeks to elucidate the relationships among physical fitness, external loads, and HRR following high-intensity exercises or performance tests in the context of five consecutive official matches within a congested training schedule. By investigating these dynamics and considering two substitution strategies, this research aims to provide a comprehensive understanding of how players' physiological responses and recovery mechanisms contribute to their performance during demanding futsal schedules.

## Methods

### Participants

Eleven elite outfield futsal players voluntarily participated in this study (age = 18.6 ± 0.7 yrs; body height = 1.7 ± 0.1 m; body mass = 64.1 ± 8.4 kg; maximum HR = 196.2 ± 8.8 bpm; VO_2max_ = 51.8 ± 3.8ml·kg^−1^•min^−1^). Participants were selected according to specific inclusion criteria: (i) consistent attendance at all sessions, (ii) absence of injuries in the two weeks leading up to the training camp, (iii) absence of injuries or illnesses throughout the two-week observation period, and (iv) exclusion of players who missed more than two training sessions. Participants signed informed consent forms and were familiar with the testing protocol. The study was approved by the institutional board of the Human Ethics Committee at the University of Taipei (protocol code UT-IRB-2018-068; approval date: 03 January 2019) and undertaken following the Declaration of Helsinki and its later amendments in 2013.

### Measures

This study followed an observational design and was conducted during a ten-day futsal training camp held in Portugal, precisely two weeks ahead of the Asian under-20 Futsal Championship final in Tabriz, Iran. The training camp encompassed various components: five friendly match days against a regional futsal club, four training days, one dedicated resting day, and two days allocated for international travel. Friendly matches were strategically scheduled on the 1^st^, 3^rd^, 5^th^, 7^th^, and 9^th^ day of the training camp. To gauge the internal and external loads on players, the exercise HR and locomotion profiles during these matches were recorded. It is important to note that data from goalkeepers were excluded from the subsequent statistical analysis.

### Design and Procedures

The players' individual anthropometric characteristics (age, body height, and body mass) and physical fitness [HR_max_, VO_2max_, maximal aerobic speed (MAS) during the 30–15 intermittent fitness test (IFT)] were measured at 4 p.m. of the initial day during a 5-day domestic training camp (registration day, 18^th^ of February 2019). The physical fitness protocol was organized as anthropometric assessments, followed by the 30–15 IFT. All players performed a 10-min dynamic warm up exercises prior to the physical fitness assessment in an indoor sports hall. An integrated HR monitor system (Polar team Pro, Polar Electro, Kemple, Finland) was used to record the HR_max_ during the 30–15 IFT. The ambient temperature and relative humidity were 27°C and 60%, respectively. One player did not perform the 30–15 IFT due to a personal schedule issue. For external load and HRR records, a Polar telemetric GPS (Polar team Pro, Polar Electro, Kemple, Finland) was used to record the HR responses and locomotion profiles during match play. Each player had their sensor attached via an HR strap at the chest level. The Polar GPS was previously tested for its reliability and accuracy, having been determined as with good inter-unit reliability for load measures, with the following ranging values: 0.63–0.99 for the intraclass correlation coefficient (ICC) and 0.6–13.8% for the typical error of measurement (TEM) for the overall load measures (Akyildiz et al., 2020).

For external loads, locomotion variables extracted for each field play were included: 1) the number of decelerations at speed of −50.00–−3.00 m/s^2^ (ND1); 2) the number of decelerations at speed of −2.99–−2.00 m/s^2^ (ND2); 3) the number of decelerations at speed of −1.99–−1.00 m/s^2^ (ND3); 4) the number of accelerations at speed of 1.00–1.99 m/s^2^ (NA1); 5) the number of accelerations at speed of 2.00–2.99 m/s^2^ (NA2); and 6) the number of accelerations at speed of 3.00–50.00 m/s^2^ (NA3).

HRR was recorded while players were substituted after each field play. Considering the variability of recovery time for each substituted player, the first 3-minute time window of HRR kinetics was calculated in this study. HRR variables included the exercise HR at field play termination (post-HR0), the differences of HR responses between field play termination and HRR at 30 s (post-HR30), HRR at 60 s (post-HR60), HRR at 120 s (post-HR120), and HRR at 180 s (post-HR180).

All sensors for GPS and HRR records were synced to a Polar team pro dock after the matches. Raw data were uploaded to a Polar cloud server and exported to a laptop for data analysis. Data were excluded for statistical analysis when the detection of locomotion profiles and HRR were lost due to physical contact between players or other vigorous physical activities during match play.

### Statistical Analysis

Descriptive statistics were presented as means ± standard deviations (SDs). The Kolmogorov-Smirnov test (> 50 samples) was used to examine data distribution for normality. A two-way mixed analysis of variance (ANOVA) was used to analyze the number of substitutions and time of HRR. The sphericity assumed was used to compare individual data points obtained from each condition.

A Pearson's correlation coefficient (*r*) was used to examine the relationships among physical fitness, external loads, and HRR measures during a congested schedule of futsal matches. The magnitude of Pearson's correlations was defined as *r* ≤ 0.1, trivial; 0.1 < r ≤ 0.3, small; 0.3 < *r* ≤ 0.5, moderate; 0.5 < *r* ≤ 0.7, large; 0.7 < *r* ≤ 0.9, very large; and *r* > 0.9, almost perfect. The data treatment and analysis were performed in the Statistica program (version 13.1; Statsoft, Inc., Tulsa, OK, USA). The level of significance was accepted at *p* < 0.05 for all analyses.

## Results

In Match 1, a significant HRR main effect was found (F_5,25_ = 357.36, *p* < 0.001, η^2^ = 0.99, very large). In Match 2, there were significant HRR main effects (F_5,20_ = 171.12, *p* = 0.001, η^2^ = 0.98, very large) and an interaction (F_20,80_ = 2.76, *p* < 0.01, η^2^ = 0.41, medium). Match 3 showed significant main effects on substitutions (F_4,24_ = 4.61, *p* < 0.01, η^2^ = 0.44, medium) and HRR (F_5,30_ = 173.26, *p* < 0.001, η^2^ = 0.97, very large). In Match 4, a significant HRR main effect emerged (F_5,15_ = 298.43, *p* < 0.001, η^2^ = 0.99, very large), and the same applied to Match 5 (F_5,10_ = 164.14, *p* < 0.001, η^2^ = 0.99, very large). All pairwise comparisons showed significance (Table 1, *p* ≤ 0.001).

**Table 1:** Heart rate recovery during a congested fixture

Variables	S 1	S 2	S 3	S 4	S 5
Match 1					
Post-HR0 (bpm)	187.5 ± 8.8	189.9 ±6.3	189.2 ± 6.3	190.4 ± 6.8	187.2 ± 6.7
Post-HR30 (bpm)	174.6 ± 14.4	174.6 ± 9.2	176.4 ± 7.8	177.5 ± 7.6	174.8 ± 10.0
Post-HR60 (bpm)	157.6 ± 13.6	156.2 ± 11.0	156.7 ± 12.0	160.5 ± 12.1	155.6 ± 14.0
Post-HR120 (bpm)	142.2 ± 11.2	137.9 ± 13.0	141.4 ± 11.1	142.2 ± 13.4	138.9 ± 10.7
Post-HR180 (bpm)	134.5 ± 11.8	130.6 ± 13.5	133.6 ± 13.1	134.4 ± 12.1	135.2 ± 6.3
Match 2					
Post-HR0 (bpm)	178.2 ± 10.5	158.9 ± 57.9	183.6 ± 9.0	177.5 ± 13.2	181.6 ± 15.0
Post-HR30 (bpm)	157.4 ± 14.6	140.5 ± 52.5	164.2 ± 16.2	156.1 ± 20.1	167.0 ± 20.2
Post-HR60 (bpm)	138.6 ± 15.6	122.3 ± 46.6	144.0 ± 16.6	133.0 ± 15.8	145.0 ± 16.0
Post-HR120 (bpm)	122.2 ± 14.0	110.3 ± 42.8	122.5 ± 13.7	117.6 ± 18.1	129.2 ± 16.0
Post-HR180 (bpm)	115.6 ± 15.4	108.9 ± 40.5	114.1 ± 10.2	112.4 ± 13.4	122.4 ± 15.6
Match 3					
Post-HR0 (bpm)	187.0 ± 6.9	190.4 ± 7.2	184.9 ± 6.1	183.3 ± 14.2	179.3 ± 15.9
Post-HR30 (bpm)	171.4 ± 10.6	177.1 ± 7.5	169.1 ± 8.2	165.4 ± 13.5	160.6 ± 20.6
Post-HR60 (bpm)	154.8 ± 11.3	159.1 ± 12.2	151.0 ± 14.1	141.6 ± 17.2	137.9 ± 12.0
Post-HR120 (bpm)	133.9 ± 11.3	133.9 ± 9.0	131.3 ± 12.8	126.4 ± 13.2	121.9 ± 10.0
Post-HR180 (bpm)	129.1 ± 12.3	127.4 ± 11.9	124.8 ± 13.4	119.4 ± 11.5	117.6 ± 11.2
Match 4					
Post-HR0 (bpm)	183.3 ± 10.4	181.9 ± 8.0	182.2 ± 6.5	185.9 ± 6.1	187.5 ± 6.9
Post-HR30 (bpm)	166.9 ± 17.5	165.8 ± 14.1	161.2 ± 11.5	167.0 ± 10.0	170.5 ± 8.6
Post-HR60 (bpm)	147.5 ± 20.4	142.8 ± 15.0	142.2 ± 13.8	145.4 ± 6.4	146.2 ± 6.5
Post-HR120 (bpm)	126.6 ± 12.7	123.1 ± 13.4	123.3 ± 9.3	128.0 ± 11.0	126.0 ± 5.3
Post-HR180 (bpm)	121.0 ± 11.7	115.0 ± 17.6	117.9 ± 9.5	122.9 ± 8.7	122.8 ± 6.5
Match 5					
Post-HR0 (bpm)	188.8 ± 7.1	187.8 ± 6.4	180.1 ± 7.4	184.0 ± 5.5	175.4 ± 7.5
Post-HR30 (bpm)	176.1 ± 7.7	172.9 ± 9.2	164.9 ± 11.7	170.2 ± 3.8	158.2 ± 12.8
Post-HR60 (bpm)	155.4 ± 14.9	152.4 ± 10.9	144.9 ± 10.1	145.8 ± 6.7	141.4 ± 10.7
Post-HR120 (bpm)	131.3 ± 18.0	135.4 ± 14.9	131.0 ± 8.7	130.2 ± 6.9	123.8 ± 10.0
Post-HR180 (bpm)	125.1 ± 14.7	125.0 ± 12.0	121.6 ± 8.4	122.7 ± 11.6	117.8 ± 8.8

Note: S: substitution; bpm, beat per minute; Post-HR, post-exercise heat rate. Significant difference was identified in all pairwise comparisons of time effect.

Pearson's correlation coefficients were calculated to examine the relationships among anthropometry, physical fitness, and HRR during a congestive schedule of futsal matches. The results revealed that correlation levels between anthropometry and HRR (Table 2) and physical fitness and HRR (Table 3) were inconsistent across all the matches and substitutions.

**Table 2:** Correlations between anthropometry and heart rate recovery during match substitutions.

	Age					Body height					Body mass				
	S1	S2	S3	S4	S5	S1	S2	S3	S4	S5	S1	S2	S3	S4	S5
Match 1															
Post-HR0 (bpm)	−0.33	−0.40	−0.55	−0.98*	0.00	0.21	0.47	−0.63	−0.84	−0.73	0.22	0.56	−0.70	−0.91	−0.88
Post-HR30 (bpm)	0.00	−0.32	0.36	−1.00*	0.00	0.09	0.55	−0.50	−0.75	−0.47	0.01	0.64	−0.61	−0.77	−0.68
Post-HR60 (bpm)	0.60	−0.25	0.58	−0.99*	0.26	0.78	0.19	−0.34	−0.73	−0.85	0.72	0.30	−0.44	−0.86	−0.95
Post-HR120 (bpm)	−0.78	−0.43	0.96*	−0.88	−0.94	−0.34	−0.03	−0.73	−0.77	−0.92	−0.31	0.03	−0.68	−0.87	−0.99
Post-HR180 (bpm)	−0.83	−0.48	0.88	−0.85	−0.40	−0.36	0.34	−0.73	−0.80	−0.99	−0.31	0.40	−0.77	−0.90	−1.00
Match 2															
Post-HR0 (bpm)	0.84	0.65	0.49	0.50	−0.38	0.22	0.75	−0.81	−0.67	0.62	0.28	0.83	−0.78	−0.74	0.40
Post-HR30 (bpm)	0.86	0.49	0.59	0.51	−0.25	0.29	0.76	−0.81	−0.78	0.43	0.33	0.83	−0.87	−0.86	0.19
Post-HR60 (bpm)	0.66	0.55	0.51	0.39	0.64	0.37	0.74	−0.63	−0.75	0.16	0.44	0.82	−0.57	−0.93	−0.10
Post-HR120 (bpm)	0.60	0.58	−0.47	0.62	0.71	0.18	0.18	−0.39	−0.78	−0.90	0.25	0.31	−0.33	−0.86	−0.98
Post-HR180 (bpm)	0.85	0.82	−0.46	−0.73	0.61	0.11	0.39	−0.60	−0.88	−0.87	0.20	0.48	−0.48	−0.95*	−0.96
Match 3															
Post-HR0 (bpm)	0.06	−0.22	0.30	−0.50	0.69	−0.03	0.87	−0.64	−0.49	0.81	−0.01	0.90	−0.57	−0.81	0.93
Post-HR30 (bpm)	0.38	0.05	0.76	−0.36	0.91*	0.80	0.79	0.03	−0.01	0.88	0.80	0.86	−0.01	−0.29	0.97
Post-HR60 (bpm)	0.43	0.46	0.20	−0.11	0.86	0.85	0.90	0.04	−0.09	0.06	0.81	0.92	0.03	−0.48	0.31
Post-HR120 (bpm)	0.15	0.28	0.20	−0.11	0.40	0.94	−0.22	−0.14	−0.47	0.36	0.91	−0.13	−0.12	−0.81	0.59
Post-HR180 (bpm)	−0.07	0.15	−0.10	0.35	0.44	0.99*	0.12	−0.37	−0.48	−0.11	0.97*	0.14	−0.31	−0.81	0.14
Match 4															
Post-HR0 (bpm)	0.25	0.51	−0.51	−0.99*	−0.18	−0.66	0.93	−0.31	−0.18	0.00	−0.61	0.88	−0.06	−0.32	−0.25
Post-HR30 (bpm)	0.49	0.35	0.57	−0.95*	0.45	−0.76	0.94	−0.19	−0.32	0.00	−0.71	0.91	0.07	−0.34	−0.25
Post-HR60 (bpm)	0.31	0.08	0.17	−0.92	−0.04	−0.77	0.85	−0.07	−0.38	0.00	−0.71	0.86	0.16	−0.35	−0.25
Post-HR120 (bpm)	0.33	0.35	−0.27	−0.68	−0.35	−0.46	0.43	−0.12	0.67	--	−0.38	0.34	0.03	0.44	--
Post-HR180 (bpm)	0.25	0.13	−0.06	−0.96*	−0.12	−0.61	−0.46	−0.24	−0.10	0.00	−0.52	−0.58	−0.30	−0.32	−0.25
Match 5															
Post-HR0 (bpm)	−0.98	0.14	1.00	−0.50	−0.15	0.23	−0.13	0.81	−0.98*	−0.62	0.16	−0.27	0.89*	−0.83	−0.41
Post-HR30 (bpm)	−0.85	−0.08	1.00*	−0.50	0.27	−0.03	0.23	0.58	−0.81	−0.25	−0.13	0.11	0.68	−0.79	0.00
Post-HR60 (bpm)	−1.00*	−0.36	0.55	−0.50	0.41	0.32	0.76	0.38	−0.85	−0.10	0.23	0.67	0.52	−0.99*	0.15
Post-HR120 (bpm)	−0.99	−1.00	0.78	--	−0.58	−0.56	0.49	0.46	−0.71	−0.09	−0.50	0.42	0.36	−0.95	−0.34
Post-HR180 (bpm)	−0.93	−1.00	0.40	−0.50	0.99	−0.62	0.35	−0.25	−0.95*	0.77	−0.55	0.27	−0.10	−0.99*	0.90

Note: S: substitution; Post-HR: post-exercise heat rate. * indicates a significant difference

**Table 3:** Correlations between physical fitness and heart rate recovery during match substitutions.

	HR_max_					VO_2max_					30–15 IFT					
	S1	S2	S3	S4	S5	S1	S2	S3	S4	S5	S1	S2	S3	S4	S5
Match 1															
Post-HR0 (bpm)	−0.87	−0.43	0.51	0.32	0.86	−0.27	−0.42	0.01	0.45	0.62	−0.31	−0.39	−0.10	0.10	0.59
Post-HR30 (bpm)	−0.20	−0.42	0.46	0.16	0.98	0.12	−0.50	0.06	0.27	0.33	0.11	−0.48	−0.05	−0.09	0.30
Post-HR60 (bpm)	−0.42	−0.58	0.46	0.40	0.75	−0.60	−0.14	−0.18	0.50	0.76	−0.60	−0.11	−0.28	0.17	0.73
Post-HR120 (bpm)	−0.65	−0.17	0.23	0.33	0.64	0.20	0.13	−0.08	0.45	0.85	0.16	0.17	−0.20	0.10	0.83
Post-HR180 (bpm)	−0.68	−0.11	0.44	0.37	0.44	0.16	−0.25	0.06	0.49	0.95	0.11	−0.20	−0.05	0.15	0.94
Match 2															
Post-HR0 (bpm)	−0.97*	−0.51	0.31	0.25	0.57	−0.42	−0.78	0.08	0.33	−0.73	−0.46	−0.78	−0.02	0.00	−0.76
Post-HR30 (bpm)	−0.98*	−0.45	0.41	0.30	0.74	−0.44	−0.82	0.28	0.42	−0.56	−0.48	−0.83	0.17	0.07	−0.59
Post-HR60 (bpm)	−0.99*	−0.51	0.12	0.56	0.90	−0.58	−0.79	−0.13	0.67	−0.31	−0.62	−0.80	−0.25	0.36	−0.34
Post-HR120 (bpm)	−0.95*	−0.72	0.20	0.29	0.67	−0.41	−0.16	−0.47	0.41	0.83	−0.45	−0.14	−0.56	0.06	0.81
Post-HR180 (bpm)	−0.91	−0.42	0.10	0.35	0.73	−0.38	−0.33	−0.33	0.50	0.78	−0.42	−0.30	−0.43	0.15	0.76
Match 3															
Post-HR0 (bpm)	−0.82	−0.19	0.18	0.87	−0.79	−0.08	−0.93	−0.19	0.95*	−0.71	−0.12	−0.94	−0.31	0.81	−0.69
Post-HR30 (bpm)	−0.81	−0.45	0.21	0.52	−0.71	−0.79	−0.83	−0.47	0.47	−0.79	−0.80	−0.84	−0.54	0.33	−0.77
Post-HR60 (bpm)	−0.56	−0.10	0.33	0.83	−0.97	−0.72	−0.95	−0.67	0.79	0.09	−0.72	−0.96*	−0.71	0.67	0.13
Post-HR120 (bpm)	−0.12	−0.83	0.30	0.89	−1.00*	−0.81	0.11	−0.60	0.95	−0.21	−0.78	0.07	−0.66	0.77	−0.18
Post-HR180 (bpm)	−0.32	−0.31	0.27	0.83	−0.92	−0.89	−0.25	−0.43	0.88	0.26	−0.87	−0.30	−0.50	0.67	0.29
Match 4															
Post-HR0 (bpm)	−0.38	0.33	−0.35	0.48	0.96	0.45	−0.95	−0.23	0.55	−0.16	0.41	−0.95*	−0.23	0.63	−0.19
Post-HR30 (bpm)	−0.25	0.18	−0.37	0.25	0.96	0.56	−0.97*	−0.43	0.35	−0.16	0.52	−0.98*	−0.43	0.39	−0.19
Post-HR60 (bpm)	−0.21	−0.09	−0.23	0.17	0.96	0.56	−0.91	−0.69	0.29	−0.16	0.52	−0.93	−0.70	0.30	−0.19
Post-HR120 (bpm)	−0.56	0.79	−0.01	0.43	--	0.19	−0.31	−0.65	0.31	--	0.14	−0.28	−0.68	0.63	--
Post-HR180 (bpm)	−0.25	0.62	0.43	0.63	0.96	0.32	0.45	−0.39	0.68	−0.16	0.29	0.43	−0.48	0.76	−0.19
Match 5															
Post-HR0 (bpm)	−0.42	0.89	−0.47	−0.06	−0.56	−0.07	0.14	−0.59	0.15	0.74	−0.08	0.14	−0.53	−0.15	0.76
Post-HR30 (bpm)	0.01	0.78	−0.46	0.09	−0.85	0.28	−0.26	−0.59	0.22	0.40	0.28	−0.27	−0.57	−0.14	0.43
Post-HR60 (bpm)	−0.18	0.77	−0.29	0.49	−0.92	−0.08	−0.70	−0.87	0.64	0.26	−0.08	−0.68	−0.89*	0.32	0.29
Post-HR120 (bpm)	−0.50	0.71	0.37	0.72	0.98	0.35	−0.37	−0.71	0.84	−0.07	0.31	−0.34	−0.72	0.61	−0.10
Post-HR180 (bpm)	0.46	0.76	−0.04	0.32	−0.84	0.44	−0.23	−0.67	0.49	−0.66	0.44	−0.19	−0.75	0.17	−0.63

Note: S: substitution; HR_max_; maximum heart rate; VO_2max_: maximal oxygen uptake; 30–15 IFT: 30–15 intermittent fitness test; Post-HR: post-exercise heat rate. * indicates a significant difference

Furthermore, Pearson's correlation coefficients were used to examine the relationships between match locomotion profiles and HRR during a congestive schedule of futsal matches (Tables 4 and 5). The numbers of moderate (NA1: 1.00–1.99 m/s^2^), moderate-to-high (NA2: 2.00–2.99 m/s^2^), and high (NA3: 3.00–50.00 m/s^2^) intensities of acceleration presented negative correlations in the last match (*r* < −0.76; *p* < 0.05).

**Table 4:** Correlations between decelerations and heart rate recovery during match substitution.

	ND1					ND2					ND3				
	S1	S2	S3	S4	S5	S1	S2	S3	S4	S5	S1	S2	S3	S4	S5
Match 1															
Post-HR0 (bpm)	0.21	−0.60	−0.16	0.26	−0.99	0.20	−0.27	−0.14	0.46	−0.99	0.02	0.36	−0.21	0.65	−0.73
Post-HR30 (bpm)	0.28	−0.70	−0.18	0.00	−0.92	0.46	−0.32	0.36	0.45	−0.94	0.16	0.24	0.43	0.79	−0.97
Post-HR60 (bpm)	0.22	−0.58	−0.28	0.18	−0.96	0.36	−0.48	0.26	0.60	−0.95	0.19	0.22	0.23	0.61	−0.62
Post-HR120 (bpm)	0.18	−0.83	−0.25	0.17	−0.87	−0.13	−0.62	−0.10	0.53	−0.85	−0.21	−0.28	−0.04	0.66	−0.42
Post-HR180 (bpm)	0.25	−0.93*	−0.06	0.25	−0.80	−0.21	−0.60	−0.08	0.52	−0.78	−0.18	−0.34	0.03	0.62	−0.30
Match 2															
Post-HR0 (bpm)	0.12	−0.94*	0.07	−0.03	−0.98	−0.12	−0.73	−0.32	0.56	−0.98	−0.15	−0.60	−0.40	0.74	−0.92
Post-HR30 (bpm)	0.33	−0.93*	0.29	0.16	−0.83	0.33	−0.71	0.07	0.51	−0.85	0.25	−0.60	−0.07	0.68	−1.00*
Post-HR60 (bpm)	0.28	−0.93*	−0.03	0.40	−0.90	−0.06	−0.73	0.20	0.62	−0.91	0.18	−0.62	0.03	0.43	−0.99
Post-HR120 (bpm)	0.32	−0.91*	−0.48	0.15	−0.93	−0.20	−0.77	−0.22	0.50	−0.91	0.14	−0.61	−0.27	0.69	−0.54
Post-HR180 (bpm)	0.21	−0.93*	−0.35	0.37	−0.94	−0.25	−0.73	−0.30	0.42	−0.93	−0.02	−0.58	−0.33	0.59	−0.56
Match 3															
Post-HR0 (bpm)	0.25	−0.94*	−0.26	0.82	−0.88	−0.04	−0.65	−0.31	0.58	−0.87	−0.08	−0.59	−0.52	−0.22	−0.44
Post-HR30 (bpm)	0.14	−0.91*	−0.37	−0.28	−0.97	0.13	−0.61	0.44	0.92	−0.96	0.04	−0.40	0.21	0.30	−0.64
Post-HR60 (bpm)	0.23	−0.92*	−0.49	0.12	−0.30	0.35	−0.50	−0.11	0.99*	−0.26	0.25	−0.47	−0.03	0.01	0.32
Post-HR120 (bpm)	−0.24	−0.62	−0.40	0.63	−0.55	0.02	−0.92*	−0.25	0.76	−0.52	−0.24	−0.67	−0.39	−0.07	0.04
Post-HR180 (bpm)	−0.18	−0.69	−0.29	0.46	−0.39	0.07	−0.74	−0.40	0.83	−0.35	−0.07	−0.79	−0.37	0.10	0.23
Match 4															
Post-HR0 (bpm)	0.12	−0.96*	−0.14	0.94	−0.91	−0.54	−0.49	−0.89*	−0.13	−0.93	−0.46	−0.60	−0.83*	−0.67	−0.98
Post-HR30 (bpm)	0.38	−0.86	−0.51	0.91	−0.99	−0.27	−0.25	−0.91*	−0.36	−0.98	−0.18	−0.40	−0.76*	−0.51	−0.71
Post-HR60 (bpm)	0.34	−0.90*	−0.68	0.89	−0.96	−0.23	−0.51	−0.75*	−0.43	−0.95	−0.17	−0.53	−0.73	−0.43	−0.61
Post-HR120 (bpm)	0.29	−0.95*	−0.58	0.33	−0.94	−0.33	−0.47	−0.66	0.05	−0.93	−0.12	−0.57	−0.66	−1.00*	−0.56
Post-HR180 (bpm)	0.02	−0.69	−0.50	0.92	−0.97	−0.37	−0.62	−0.10	0.05	−0.98	−0.36	−0.93*	−0.16	−0.74	−0.92
Match 5															
Post-HR0 (bpm)	0.24	−0.84	−0.46	0.54	0.31	−0.22	−0.60	−0.19	−0.21	0.34	−0.19	−0.83	−0.58	0.52	0.81
Post-HR30 (bpm)	0.02	−0.86	−0.58	0.04	0.71	−0.24	−0.56	−0.02	0.36	0.74	−0.43	−0.82	−0.45	0.82	0.99
Post-HR60 (bpm)	0.09	−0.97*	−0.79*	0.52	−0.34	0.04	−0.33	−0.21	0.47	−0.31	−0.21	−0.54	−0.53	0.42	0.27
Post-HR120 (bpm)	0.19	−0.92*	−0.48	0.75	−1.00	0.05	−0.25	−0.29	0.51	−1.00*	−0.13	−0.38	−0.56	0.07	−0.86
Post-HR180 (bpm)	−0.21	−0.92*	−0.66	0.58	−0.91	0.06	−0.38	−0.31	0.22	−0.89	−0.40	−0.49	−0.41	0.47	−0.49

Note: S: substitution; ND1: number of decelerations (−50.00–−3.00 m/s^2^): ND2: number of decelerations (−2.99–−2.00 m/s^2^): ND3: number of decelerations (−1.99–−1.00 m/s^2^); Post-HR: post-exercise heat rate. * indicates a significant difference.

**Table 5:** Correlations between accelerations and heart rate recovery during match substitution.

	NA1					NA2					NA3					
	S1	S2	S3	S4	S5	S1	S2	S3	S4	S5	S1	S2	S3	S4	S5
Match 1															
Post-HR0 (bpm)	0.28	0.56	−0.23	−0.93	−0.45	−0.01	−0.27	−0.06	0.44	−0.94	0.36	−0.41	−0.62	−0.99*	0.30
Post-HR30 (bpm)	0.49	0.47	0.31	−0.80	−0.83	0.07	−0.40	0.33	0.24	−0.99	0.46	−0.50	−0.59	−0.97*	−0.22
Post-HR60 (bpm)	0.49	0.40	0.11	−0.90	−0.31	−0.01	−0.28	0.20	0.48	−0.87	0.27	−0.20	−0.67	−1.00*	0.43
Post-HR120 (bpm)	−0.08	0.06	−0.14	−0.89	−0.07	−0.04	−0.70	−0.03	0.43	−0.73	0.27	−0.28	−0.80*	−1.00*	0.64
Post-HR180 (bpm)	−0.15	0.01	−0.01	−0.93	0.05	0.06	−0.81	0.05	0.48	−0.64	0.35	−0.46	−0.75	−1.00*	0.73
Match 2															
Post-HR0 (bpm)	0.00	−0.32	−0.40	−0.78	−0.72	−0.08	−0.90*	−0.15	0.29	−1.00*	0.22	−0.50	−0.56	−0.97*	−0.04
Post-HR30 (bpm)	0.45	−0.34	−0.15	−0.89	−0.92	0.18	−0.89*	0.24	0.40	−0.94	0.52	−0.54	−0.58	−0.99*	−0.40
Post-HR60 (bpm)	0.17	−0.35	−0.22	−0.97*	−0.87	0.17	−0.90*	0.24	0.66	−0.98	0.38	−0.50	−0.89*	−0.97*	−0.28
Post-HR120 (bpm)	0.02	−0.33	−0.34	−0.88	−0.21	0.24	−0.88	−0.26	0.39	−0.82	0.41	−0.40	−0.73	−0.99*	0.53
Post-HR180 (bpm)	−0.07	−0.27	−0.42	−0.96*	−0.24	0.11	−0.89*	−0.26	0.50	−0.84	0.32	−0.44	−0.82*	−0.97*	0.50
Match 3															
Post-HR0 (bpm)	0.05	−0.33	−0.53	−0.83	−0.10	0.03	−0.91*	−0.28	0.97*	−0.75	0.33	−0.62	−0.36	−0.61	0.62
Post-HR30 (bpm)	0.28	−0.18	−0.01	−0.40	−0.33	−0.07	−0.79	0.24	0.38	−0.88	0.23	−0.65	−0.66	−0.68	0.42
Post-HR60 (bpm)	0.52	−0.24	−0.03	−0.58	0.63	0.03	−0.85	−0.22	0.73	−0.07	0.28	−0.76	−0.46	−0.69	0.99
Post-HR120 (bpm)	0.17	−0.62	−0.42	−0.86	0.39	−0.46	−0.61	−0.31	0.94	−0.34	−0.21	−0.15	−0.70	−0.75	0.92
Post-HR180 (bpm)	0.28	−0.70	−0.36	−0.87	0.55	−0.37	−0.76	−0.36	0.85	−0.16	−0.20	−0.39	−0.71	−0.85	0.98
Match 4															
Post-HR0 (bpm)	−0.56	−0.30	−0.72	−0.28	−0.85	−0.03	−0.96	−0.74	0.63	−0.98	0.17	−0.73	−0.15	0.16	−0.24
Post-HR30 (bpm)	−0.35	−0.16	−0.66	−0.27	−0.42	0.29	−0.82	−0.87*	0.45	−0.92	0.55	−0.89	−0.23	0.17	0.33
Post-HR60 (bpm)	−0.30	−0.31	−0.66	−0.27	−0.29	0.27	−0.85	−0.80*	0.39	−0.87	0.56	−0.74	−0.37	0.16	0.45
Post-HR120 (bpm)	−0.27	−0.16	−0.60	0.43	−0.24	0.22	−0.96*	−0.68	0.33	−0.84	0.46	−0.57	−0.47	0.66	0.50
Post-HR180 (bpm)	−0.37	−0.67	−0.19	−0.30	−0.73	−0.03	−0.88*	−0.17	0.74	−1.00*	0.27	−0.25	−0.56	0.11	−0.05
Match 5															
Post-HR0 (bpm)	−0.18	−0.51	−0.61	−0.77	0.96	−0.04	−0.96*	−0.37	0.21	0.52	0.07	−0.42	0.18	−0.61	0.88
Post-HR30 (bpm)	−0.26	−0.52	−0.57	−0.81	0.98	−0.27	−0.98*	−0.26	0.20	0.86	−0.06	−0.52	−0.01	−0.95	0.57
Post-HR60 (bpm)	0.08	−0.14	−0.63	−1.00*	0.59	−0.19	−0.98*	−0.43	0.65	−0.11	0.12	−0.74	−0.33	−0.94	0.99
Post-HR120 (bpm)	0.08	0.06	−0.50	−0.96*	−0.63	−0.01	−0.90*	−0.39	0.86	−0.99	0.37	−0.64	−0.18	−0.78	0.08
Post-HR180 (bpm)	0.02	−0.06	−0.50	−0.97*	−0.16	−0.35	−0.93*	−0.40	0.53	−0.79	0.12	−0.56	−0.67	−0.85	0.57

Note: S: substitution; NA1: number of accelerations (1.00–1.99 m/s^2^); NA2: number of accelerations (2.00–2.99 m/s^2^); NA3: number of accelerations (3.00–50.00 m/s^2^); Post-HR: post-exercise heat rate. * indicates a significant difference.

## Discussion

The present study examined relationships among physical fitness, external loads, and HRR, considering the number of substitutions during a congested-fixture futsal training camp. The main findings revealed that the overall observed correlations among anthropometry, physical fitness, and HRR were inconsistent across all the matches and substitutions. The moderate-to-high intensity of acceleration presented the relevant correlations in the last match of the congested fixed overseas training camp.

The results of the present study indicate that the time interval used to measure HRR after a period of match congestion can significantly impact the values obtained. Indeed, HRR has been extensively studied as an indicator of cardiovascular fitness and autonomic function in team sports (Daanen et al., 2012). It is generally accepted that faster HRR following exercise/match is associated with better cardiovascular health and performance (Daanen et al., 2012). However, the interval of HRR measurements after a match should be considered with previous investigations suggesting measuring HRR at 30 s to three minutes post-exercise (Rave et al., 2018). Differences in HRR between the different recovery time points after the field play may be attributed to various physiological and metabolic factors. During a match, the body undergoes significant physical stress and metabolic demands, accumulating metabolites and depleting energy stores (Bishop et al., 2003; Impellizzeri et al., 2004). The extent of this stress can vary depending on various factors, including the intensity and duration of the match, the fitness level of athletes, and their physiological responses (Krustrup et al., 2006).

Considering the relationships between physical fitness and HRR, a previous study conducted on ninety-two youth soccer players showed increased HRR with improved repeated sprint ability (Buchheit et al., 2012). Also, a study conducted on fifty-nine male and female athletes from other indoor sports found a significant relationship between the 30–15 IFT and HRR (Buchheit, 2008). Although the studies mentioned above reported similar significant relationships between different physical fitness measures and HRR, they did not consider match participation, which makes comparisons difficult. Conversely, another study on eighty-four collegiate athletes showed significant relationships between VO_2max_ performance and Post-30 HRR (Watson et al., 2017). The findings mentioned above are promising; however, it is essential to note that the research on athletes is limited, and the methodologies used to collect HRR data are heterogeneous.

In respect to the relationships between external loads and HRR, no significant correlations between HRR and increases in maximal speed were previously reported, which is in concordance with our findings (Buchheit et al., 2012). However, the lack of relationships between maximal speed and HRR was expected as HRR measures cardiovascular fitness and may not necessarily reflect improvements in power or strength-related variables. HRR reflects the parasympathetic reactivation of the autonomic nervous system following exercise, which can indicate cardiovascular health (Daanen et al., 2012). Conversely, improvements in power or strength-related variables must be assessed through measures such as maximal strength, power output or velocity, which are more specific to neuromuscular adaptations (Daanen et al., 2012).

The present study revealed negative correlations between high-intensity decelerations/accelerations and HRR. A greater cardiorespiratory capacity was previously suggested to allow players to produce a higher volume of low- and high-intensity accelerations during a match (Gabrys et al., 2020; Osgnach et al., 2010). Positive correlations between decelerations/accelerations and HRR should be expected. For instance, a recent study found a significant correlation between HRR and accelerations during SSGs (Reinhardt et al., 2020). Specifically, players who had higher accelerations during SSGs also had faster HRR after SSGs. However, this was not the case in the present study, where negative correlations were found (i.e., a higher number of accelerations implied slower HRR). This finding may be a reflection of the presence of fatigue.

The present study has some limitations that need to be explicitly stated. The main limitation is the small sample size. This fact limits the generalizability of the findings to a larger population. The very short period of the observations is another limitation, which means that it remains to be seen whether the observed relationships would remain over a more extended time or if a more consistent pattern of correlations would appear. Another limitation includes the potential confounding factors, as we did not control for other factors that could influence HRR, such as age, sex, and the fitness level, which may have impacted the results. Controlling for other influencing factors, including the individuals' position in the HRR measurement, is paramount to ensure unbiased values (Michaelson et al., 2019). Additionally, future research should encompass a holistic approach by investigating concurrent physiological and psychological markers of recovery. It has been proposed that HRR is assessed with other measures, such as muscle strength, which may be relevant for future studies.

This study's findings could be translated into actionable strategies for players’ recovery. Beyond solely relying on HRR as a measure, it is crucial to emphasize comprehensive recovery strategies. Coaches and practitioners could implement multifaceted interventions, incorporating nutrition, sleep hygiene education, and exposure to cold water immersion protocols (Nédélec et al., 2012, 2013). By developing and promoting effective recovery strategies, the futsal community can better equip athletes to endure the demands of congested schedules and achieve peak performance consistently. Furthermore, HRR can be used as a non-invasive and objective measure to monitor the training load of athletes. By tracking HRR during and after training sessions, coaches can adjust training intensity to optimize athletes’ performance and prevent overtraining.

## Conclusions

The overall observed correlations between physical fitness measures and HRR may have been influenced by uncontrolled factors across all the match play and recovery of cardiac responses during match substitutions. The HRR measures may be affected by fatigue caused by the accumulation of accelerations throughout a congested fixture during a futsal training camp. Although HRR measures provide insights into physical fitness and acceleration activities, it is suggested that coaches consider fatigue as it may be a significant factor influencing HRR values, at least during congested fixtures.
